# Cognitive Computational Neuroscience of Language: Using Computational Models to Investigate Language Processing in the Brain

**DOI:** 10.1162/nol_e_00131

**Published:** 2024-04-01

**Authors:** Alessandro Lopopolo, Evelina Fedorenko, Roger Levy, Milena Rabovsky

**Affiliations:** Department of Psychology, University of Potsdam, Potsdam, Germany; Department of Brain and Cognitive Sciences, Massachusetts Institute of Technology, Cambridge, MA, USA; McGovern Institute for Brain Research, Massachusetts Institute of Technology, Cambridge, MA, USA

## INTRODUCTION

From the inception of large-scale deep learning models to the development of cognitively inspired artificial neural networks (ANNs), computational modeling has ushered in a new era of exploration into language processing within the human brain. This special issue serves to showcase cutting-edge research in the field, all united by a common interest in employing computational models as a tool for generating and testing theories, including through methodological innovations. These studies not only underscore the remarkable progress achieved by the computational cognitive neuroscientific approach but also shed light on the immense potential that this dynamic discipline holds, promising groundbreaking insights into the mechanisms that underpin our language capacity.

The goal of neurobiology of language is to identify the neural substrates of linguistic computations and representations. This pursuit takes inspiration from a broad range of disciplines (e.g., cognitive science, linguistics, neuroscience, and neurophysiology) and domains (e.g., vision, memory, attention) to generate hypotheses that are tested against neurobiological experimental observations. However, despite considerable efforts, attempts to connect abstract theoretical constructs and the concrete properties of the human brain have encountered significant challenges, partly because it is unclear how to map between the “part list” of cognition and that of neurobiology—the “mapping problem” (Embick & Poeppel, 2015; Poeppel, 2012). The difference of these two domains necessitates a nuanced and interdisciplinary approach that respects their inherent complexities.

Computational modeling approaches can bridge the ontological gap between cognition and neurobiology by providing a means of transforming hypotheses into implemented models and stimuli into numeric descriptors. This advantage holds for not only advanced deep learning models and neural encoding analysis methods, but also more traditional tools, such as parsers that return syntactic trees, language models that provide probabilistic descriptions of stimuli (e.g., surprisal) or make quantitative predictions about processing difficulty, or traditional vector-space models based on distributional semantics. In other words, computational models transform verbal hypotheses into numeric representations or measures that can be mapped onto neural (or behavioral) data. The papers featured in this issue are unified by their common reliance on computational models of language processing in order to address the challenge of linking theoretical constructs to neural data.

## MODELS AND METHODS

In this section, we provide a concise, accessible overview of key computational concepts (language models and word embeddings) and methods which are, with all the due differences and peculiarities, integral to many of the papers featured in this special issue.

*Word embeddings*, also known as word vector representations, are numerical representations of words in a high-dimensional vector space where the relative positions of words capture their semantic relationships. These representations are typically generated using machine learning models, such as Word2Vec, GloVe, FastText, or even large-scale language models (LLMs) like GPT-2. These models learn to map words to continuous vector spaces through co-occurrence based training using large text corpora, in line with the distributional-semantics hypothesis (Landauer & Dumais, 1997; Sahlgren, 2008), where words with similar meanings occur in similar linguistic contexts and therefore have similar vector representations (or “embeddings”). Word embeddings can be used for various natural language processing (NLP) tasks.

A *language model* is a computational system used in NLP to extract information from text data and generate human language. It predicts words or sequences of words based on the preceding or surrounding context in a given text. Language models can be implemented in different ways, such as n-grams, probabilistic symbolic grammars, recurrent neural networks (RNNs), and transformer models. N-grams are a basic form of a language model that consider a fixed number of preceding words when making predictions (e.g., the preceding two words for a 3-gram model; Jurafsky & Martin, 2009). They do not explicitly model hierarchical structure and cannot capture long-range dependencies. Probabilistic context-free grammars (PCFGs; Grenander, 1967) explicitly model hierarchical structure and thus can capture long-distance dependencies. In their most common forms, however, n-gram and PCFG models do not use the similarities among different words and among different contexts to generalize their predictions. As a result, they have been mostly superseded in modern NLP work by neural network-based models, which excel in this kind of generalization. RNNs, first applied to language by Elman (1990), represent context with a high-dimensional embedding representation (similar to word embeddings) that is updated as each successive input word is processed. Like n-gram models, RNNs do not explicitly model hierarchical structure, but they can implicitly capture such structure, including long-range dependencies, through their embedding representation of the context. One RNN variant, the long short-term memory (LSTM; Hochreiter & Schmidhuber, 1997) model, has proven especially good at modeling long-range dependencies and as a result is the most widely used recurrent architecture for natural language. However, the most popular and widespread language modeling architecture today is the transformer, which instead of recurrence uses an “attention” mechanism (Vaswani et al., 2017), which allows the model to combine embedding representations across the input sequence simultaneously. Like RNNs, transformers do not explicitly represent hierarchical structure or long-distance dependencies, but with modern computing machinery they can operate over a very large context window (thousands or tens of thousands of tokens), and in practice they model long-distance dependencies very well. The transformer architecture underlies the well-known BERT (bidirectional encoder representations from transformers) and GPT (generative pre-trained transformer) models, which have achieved state-of-the-art results on NLP tasks and captured public attention due to their ability to generate fluent and coherent text. Symbolic grammar-based mechanisms can also be combined with RNN or transformer architectures (Dyer et al., 2016), and the results have been argued to improve grammatical performance (Kuncoro et al., 2017; Qian et al., 2021; Sartran et al., 2022) and predictive power for human brain responses (Brennan et al., 2020), at least for earlier versions of language models.

*Neural encoding* has emerged as one of the main approaches to studying the relationship between computational models and the human brain; other prominent approaches include decoding and representational similarity analysis (Kriegeskorte & Douglas, 2018; Kriegeskorte et al., 2008; Naselaris et al., 2011). In general terms, neural encoding refers to a set of techniques that utilize machine learning algorithms to predict a neural signal (although a similar approach can be used for predicting behavioral measures) based on a set of computational descriptors of the stimuli that were used to elicit the signal. In the field of computational cognitive neuroscience of language, such stimulus descriptions are often obtained from language models (e.g., ANN models such as RNNs or transformers) that have been trained on various language tasks, as described above. The procedure involves fitting a linear regression model to predict neural data (e.g., voxel-level responses in fMRI) from computational stimulus descriptors (e.g., word embeddings from some layer of a deep neural network language model for the same stimuli as were presented to human participants) using a subset of the stimuli. The accuracy of the encoding model is then evaluated by comparing the predicted neural activity to the actual recorded neural activity for the left-out stimuli and computing some measure of divergence.

## OVERVIEW OF THE SPECIAL ISSUE

The articles in this special issue introduce or leverage insights from novel or well-established language-processing models. One distinctive dimension corresponds to the motivation behind the employed models. Some studies utilize cognitively motivated architectures explicitly designed to mimic aspects of human language processing based on pre-existing theories. In contrast, other studies leverage large language models (LLMs) developed by engineers for NLP applications. These LLMs are employed to obtain either rich high-dimensional representations or measures of performance on some language tasks. It is worth noting, however, that even though LLMs were developed for NLP applications, they are originally based on parallel distributed processing principles that were developed to explain information processing in the brain (Rumelhart et al., 1986).

The first four articles (Kauf et al., Hosseini et al., Antonello & Huth, and Jain et al.) employ neural encoding techniques to explore the connection between representations (i.e., activation patterns) generated by computational models and patterns of activity in the human brain or behavioral patterns related to language processing.

Kauf and colleagues investigate what linguistic features of a stimulus influence the relation between language models’ representations and human brain activity in the language network. The authors used an fMRI dataset of brain responses to English sentences and systematically manipulated the stimuli for which model representations were extracted. They perturbed the sentences’ word order, removed different subsets of words, or replaced sentences with different sentences, to understand what aspects of linguistic stimuli contribute to model-to-brain similarity. Whereas removing or replacing content words substantially reduced model-to-brain similarity, removing function words, or perturbing the word order did not, suggesting that lexical semantic content, not syntactic structure, is the main contributor to model-to-brain similarity of fMRI responses in the language network.

The article by Hosseini and colleagues investigates the impact of the amount of training data on the ability of ANNs, specifically GPT-2 models, to predict brain activity and capture human neural and behavioral responses to language. They use two approaches to examine this relationship. They evaluate GPT-2 models trained on different amounts of data and at different stages of training against neural and behavioral benchmarks. The results reveal that models trained on a developmentally plausible amount of data (100 million tokens) already achieve near-maximal performance in capturing human neural and behavioral responses to language, which suggests that a massive amount of training that is critical for achieving state-of-the-art performance on some NLP benchmarks is not essential for the model to arrive at human-like representations.

Antonello and Huth critically examine the evidence for the predictive nature of linguistic processing as provided by encoding models. In particular, the positive relationship between a model’s performance on the next-word prediction task and its ability to capture human neural responses has been taken as evidence for the predictive nature of language processing in humans (e.g., Schrimpf et al., 2021). Antonello and Huth question this interpretation and propose instead an alternative hypothesis whereby the effectiveness of models in encoding fMRI data depends on how generalizable their representations are across models and tasks. Indeed, they measure the ability of models’ representations to predict one another (transfer) and find a strong correlation between this measure and encoding performance. The authors emphasize that their findings do not overturn the general idea that language processing is predictive, only that the relationship between model performance and its similarity to human neural data warrants more caution.

The article by Jain and colleagues introduces a new methodological approach for neuroscience based on neural data prediction using deep learning-based encoding models. This novel paradigm aims to combine the advantages of controlled experiments and studies that use naturalistic stimuli, resulting in more interpretable and generalizable results. Their approach involves (a) training an encoding model using a large naturalistic fMRI dataset, and then (b) simulating brain activity patterns that are expected from controlled stimulation by feeding stimuli to a neural encoding model trained on mapping naturalistic model representations and brain data. This approach offers several advantages, including experimental flexibility and prototyping, as demonstrated by four examples of simulating prior language neuroscience experiments.

The subsequent three papers (Michaelov et al., Lopopolo & Rabovsky, and Huber et al.) investigate the N400 event-related potential (ERP) component (Kutas & Hillyard, 1980). The N400 is a negative ERP component observed around 400 ms after the presentation of a potentially meaningful stimulus such as a word. In linguistic contexts, the amplitude of the N400 is influenced by several variables, including congruency with the preceding context, semantic relatedness to words in the context, word repetition, and cloze probability. Despite extensive research, an ongoing debate persists regarding the cognitive processes reflected in the amplitude of the N400 component. Core linguistic processes that play a key role in this debate include lexical-semantic access, semantic integration, and prediction at various levels. All three papers introduced here relate the N400 component to some aspect of predictive processing. However, they offer alternative interpretations of the specific predictive processes underlying this component based on modeling experiments using variables derived from models.

Michaelov and colleagues investigated whether the amplitude of the N400 component is best explained by stimulus predictability, by the similarity of the stimulus to the preceding context, or both. Word predictability was estimated by word surprisal (i.e., the negative logarithm of the conditional probability of a word in context; Frank et al., 2015; Levy, 2008), whereas cosine similarity between static word embedding representations was used to estimate semantic similarity. Their analyses indicate that surprisal outperforms semantic similarity in explaining the N400 amplitudes elicited by sentences in which the final word varies in predictability, plausibility, and semantic relationship to the most likely sentence completion. The authors suggest that this result is in line with lexical predictive coding theories and that various previously reported N400 effects can be attributed to word predictability.

The article by Lopopolo and Rabovsky modeled the N400 amplitude using (a) semantic update, that is, the difference in the internal representation of a neural network model predicting sentence meaning (the sentence gestalt model) before and after the presentation of a new stimulus (Rabovsky et al., 2018), and (b) lexical surprisal obtained from a next-word prediction LSTM language model trained on the same data, keeping model size and architecture as similar as possible. The authors find that semantic update and surprisal independently contribute to N400 amplitudes, which suggests that two distinct yet closely related subprocesses may underlie the N400 component during sentence processing.

Finally, starting from the observation that model-based surprisal predicts ERPs such as the N400, Huber and colleagues assess how surprisal predicts N400 effects during the processing of verb-final sentences in German, Basque, and Hindi. They find that N400 amplitudes are best predicted when model-based surprisal is complemented by an “Agent Preference principle” that implements a role-assignment reanalysis mechanism in case of interpretation failure. According to the authors, this finding implies that the human language processing system may not solely depend on predictive processes, as many language models do, but may additionally utilize reanalysis mechanisms based on linguistic knowledge of thematic roles.

Sugimoto and colleagues examine the ability of syntactic mechanisms in neural networks to generate surprisal values that predict patterns of fMRI activity. Similarly to Kauf and colleagues, the authors tackle the interplay between syntactic and semantic processing in AI models and the human brain. In particular, the article investigates whether recurrent neural network grammars (RNNGs), which explicitly incorporate a syntactic processing mechanism, better explain the activity in language-related brain regions as compared to language models trained to predict the next word in sequence. The results obtained on an fMRI dataset collected while participants read newspaper articles in a head-final left-branching language (Japanese) demonstrated that RNNGs outperformed sequential models in some inferior frontal and temporoparietal brain areas.

The article by Fitz and colleagues instead offers a general perspective on the role of computational modeling in language sciences in general and neurobiology of language specifically. It proposes a neurobiologically informed causal modeling approach to understand the neural mechanisms that implement language processing. Neurobiological causal models are meant to provide a mechanistic description of language processing directly grounded in the characteristics of the neurobiological substrate. This approach can potentially shed new light on the neurobiological basis for language, long-term storage in the mental lexicon, and combinatorial processing in sentence comprehension.

The final article, by Uchida and colleagues, examines the encoding of event knowledge across various model classes and the relevance of this knowledge to human language processing, with a focus on inferential processing. They examine how event knowledge is encoded in word embeddings derived from both static models (word2vec/GloVe) and contextual models (BERT/RoBERTa) and relate these representations to various forms of human inference processing, including automatic and strategic inferences as described in the psychological literature. The authors suggest that these findings may assist in predicting and interpreting specific neurophysiological markers associated with human inferential processing.

Together, the articles assembled in this special issue showcase the progress in the emerging field of cognitive computational neuroscience of language and highlight the potential of computational modeling approaches to bridge the gap between cognitive theories and neurobiology through computationally explicit models of linguistic representations and computations.
